# Synthesis of core–shell copper–graphite submicronic particles and carbon nano-onions by spark discharges in liquid hydrocarbons

**DOI:** 10.1038/s41598-021-87222-x

**Published:** 2021-04-06

**Authors:** X. Glad, J. Gorry, M. S. Cha, A. Hamdan

**Affiliations:** 1 Groupe de Physique des Plasmas, Département de Physique, Université de Montréal, 1375 Avenue Thérèse-Lavoie-Roux, Montréal, QC H2V 0B3 Canada; 2 Physical Science and Engineering Division (PSE), Clean Combustion Research Center (CCRC), King Abdullah University of Science and Technology (KAUST), Thuwal, 23955 Saudi Arabia

**Keywords:** Materials science, Nanoscale materials

## Abstract

Spark discharge in hydrocarbon liquids is considered a promising method for the synthesis of various nanomaterials, including nanocomposites. In this study, copper–carbon particles were synthesized by generating spark discharges between two Cu electrodes immersed in heptane, cyclohexane, or toluene. The synthesized particles were characterized using scanning electron microscopy, high-resolution transmission electron microscopy, and selected area electron diffraction. Overall, two families of particles were observed: Cu particles (diameter < 10 nm) embedded in a carbon matrix and submicrometric Cu particles encapsulated in a carbon shell. The obtained results indicate that the size distribution of the Cu nanoparticles and the degree of graphitization of the carbon matrix depend on the liquid. Indeed, discharges in heptane lead to Cu particles with diameters of 2–6 nm embedded in a carbon matrix of low graphitization degree, while discharges in toluene result in particles with diameters of 2–14 nm embedded in carbon matrix of high graphitization degree. Based on the obtained experimental results, it is proposed that the Cu nanoparticles are produced in the plasma core where Cu (evaporated from the electrode surface) and carbonaceous species (decomposition of the liquid) are present. When the plasma hits the electrode surface, hot (thousands of Kelvin) Cu particles are ejected from the electrode, and they propagate in the liquid. The propagation of the hot particles in the liquid results in the local evaporation of this liquid, which leads to the formation of a C-shell around each Cu particle. In few cases where the shape of the Cu particle is not spherical, carbon nanoonions are detected between the C-shell and the Cu core. These nanoonions are supposedly formed under the effect of the fluid vortices generated close to the particle surfaces when these latter are ejected into the liquid.

## Introduction

Electrical discharge in liquid is an efficient, cost-effective, and ecological technique that has great potential for use in the production of nanomaterials with complex structure^1^. This technique basically relies on the ignition of a discharge (spark or arc) between two electrodes immersed in a dielectric liquid. In 2001, Sano et al.^1^ first reported the formation of carbon onions by initiating discharges between two graphite electrodes in water. Since then, a myriad of different nanostructures had been synthesized by either changing the electrode material (titanium^2^, gold, silver^3^, lead^4^, aluminum^5^, etc*.*) or the liquid solution (e.g., heptane^6^ and other liquid hydrocarbons (HCs)^7^, liquid nitrogen^5^). Using copper electrodes in water, Glad et al.^8^ synthesized various Cu-based nanostructures whose oxygen content can be controlled by adjusting (adding HCl) the electrical conductivity of water. Other studies have demonstrated that discharges between metal electrodes in HC liquids can produce nanocomposite materials such as metal nanoparticles embedded in an amorphous matrix^9^.

Considering their great potential for use in technological applications, such as magnetic storage media, nanofluids, catalysts, etc., researchers have developed various methods for the synthesis of core–shell particles (e.g., particles of TiO_2_ with C-shell^10^ and Cu with ZnO-shell^11^), including physical- and chemical-based techniques^12–14^. For example, C-encapsulated Cu nanoparticles (NPs) were produced using complex techniques, such as (i) CVD graphene growth on Cu NPs followed by cold pressing and sintering at 600–750°C^15^, (ii) magnetron DC sputtering of metal targets coupled with RF capacitive plasma^16^ followed by a post-synthesis purification phase^12,13^, (iii) reduction of Cu^2+^ ions and single-layer graphene oxide nanosheets^17^, and (iv) decomposition of copper acetate and graphene nanoplatelets^18^. Compared to these techniques, discharge in liquid is simpler and leads to higher yields^9^, since no prepreparation or postprocessing steps are required, and the particles are instantaneously produced. However, the process of the particle formation by discharges in liquid remains unclear, partly due to the complex multidisciplinary and multiscale phenomena induced by the generation of plasma in a liquid environment comprising molten metal droplets.

In this work, a pulsed high-voltage power source was used to ignite spark discharges between two copper electrodes immersed in various liquid HCs, namely heptane, cyclohexane, and toluene. The synthesized materials in the different liquids were collected and analyzed by electron microscopy techniques. The results show that these materials include Cu NPs embedded in a carbonaceous matrix and micrometric Cu particles with C-shell.

## Results

**Figure 1:**
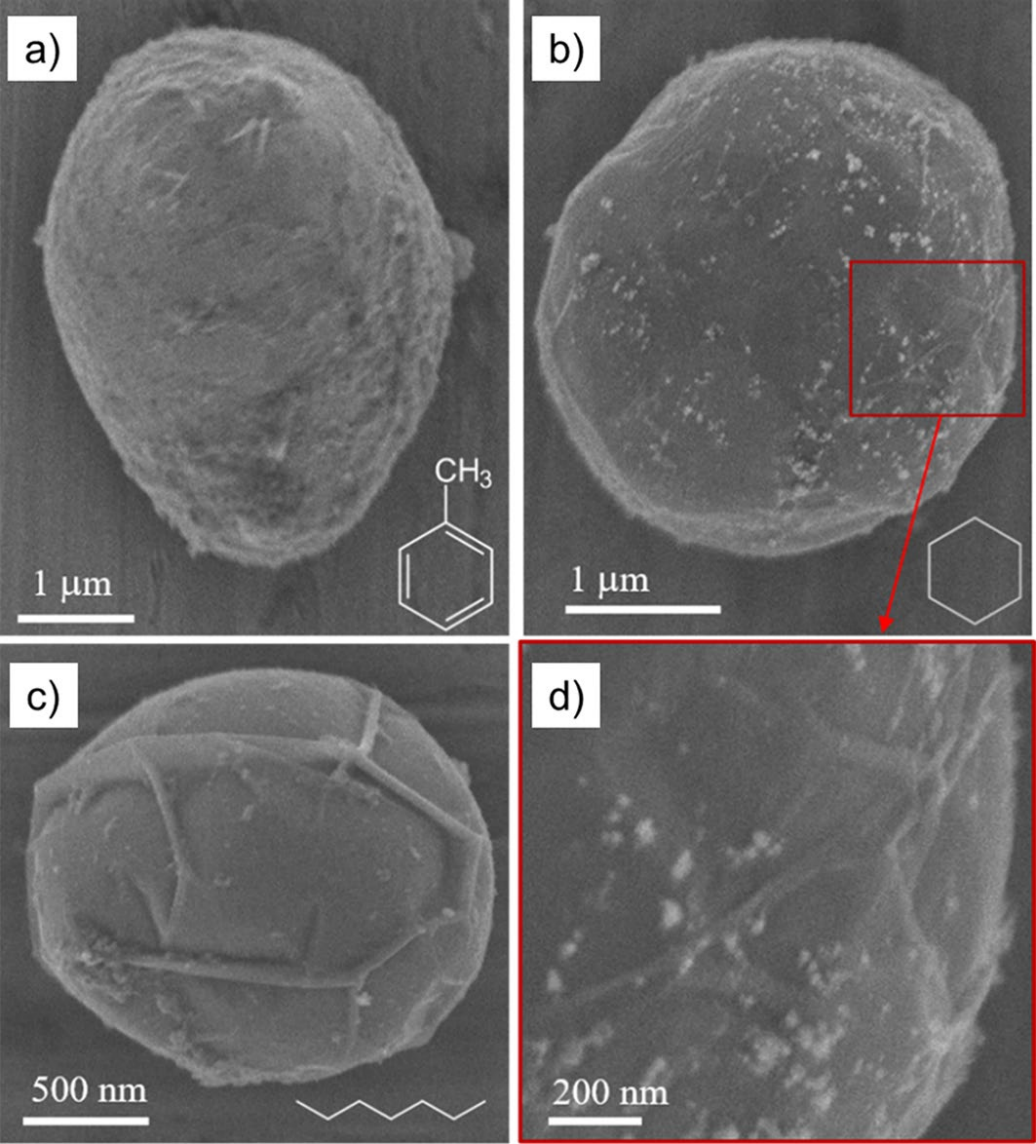
SEM images of typical microparticles collected on the SS substrates and synthesized by discharges sustained in (**a**) toluene, (**b**) cyclohexane, and (**c**) heptane. (**d**) Shows a magnification of the surface feature of (**b**) showing nanoparticles.

The setup, detailed in the *Materials and methods* section, lead to the formation of various C- and Cu-based materials. Using scanning electron microscopy (SEM) at 3 kV (penetration depth: ~ 200 nm in graphite, ~ 50 nm in Cu^19^), we investigated the Cu particles collected in all tested liquids. The surface of the stainless steel (SS) substrate (on which a liquid droplet was evaporated) is generally covered by NPs as well as by micrometric particles. The former NPs are fully observed by transmission electron microscopy (TEM), while typical microparticles synthesized in toluene, cyclohexane, and heptane are shown in Fig. 1. The surface of the particles often exhibits a folded structure, which might indicate the presence of a C-shell around a Cu core. Additionally, nanometric aggregates (similar to those found on the substrate) can be identified on the surface of the micrometric particles.

**Figure 2:**
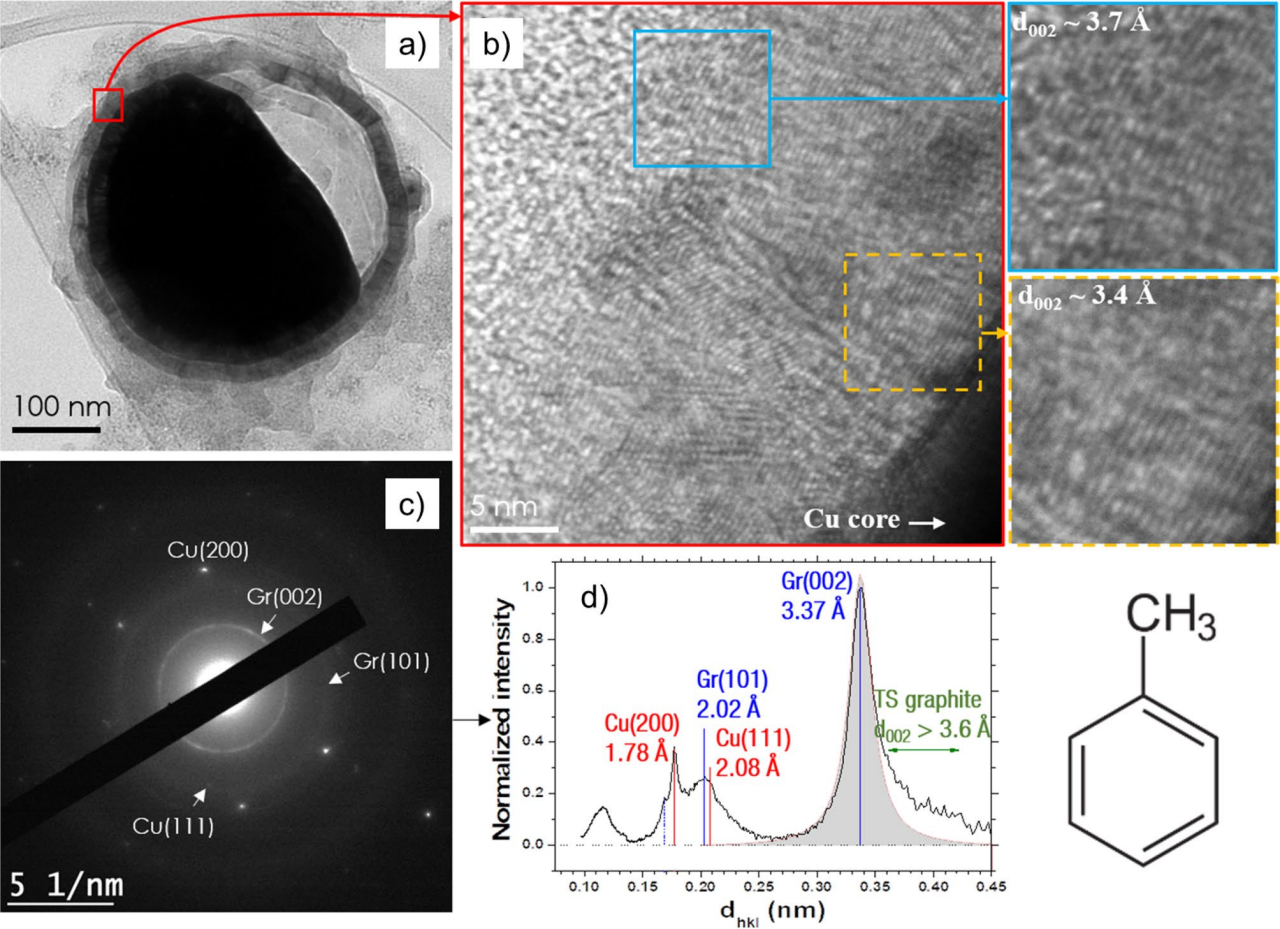
(**a**) High-resolution image of a submicronic particle synthesized by discharges in toluene. (**b**) HRTEM image of the red inset of (**a**). (**c**) SAED pattern from (**a**) exhibiting Cu (200) reflections and Gr (002) and (100) rings. (**d**) A radially integrated profile permits to highlight Cu (111) reflections. The dotted blue line corresponds to the second-order Gr (004) reflections.

Figure 2 displays TEM images of a submicrometric particle synthesized in toluene. The particle is composed of an ellipsoidal core (~ 250 nm × 350 nm) surrounded by a spherical shell (~ 390 nm of diameter). Energy Dispersive Spectroscopy (EDS) on the particle (supplementary data; Figure S1a) reveals that it is mainly composed of Cu and C; only traces of O are detected. Figure 2b presents a high resolution TEM (HRTEM) image of the C-shell, which comprises about 60 (002) graphite planes. The blue and dashed orange insets of Fig. 2b highlight the interplanar distance (d_002_) in both regions near to and far from the Cu core, with d_002_ near the Cu core being smaller than the d_002_ measured far from the core (3.4 versus 3.7 Å). A significant increase in stacking faults is also observed in the blue inset (i.e., far from the core), experiencing a substantial decrease in degree of graphitization. Figure 2c depicts the selected area electron diffraction (SAED) pattern of the whole core–shell particle. The Cu core appears as singled-out Cu(200) reflections, evidencing the high degree of crystalline order of the core, while the shell shows rings for Gr(002) and Gr(101), demonstrating the numerous orientations of the graphene planes that form the shell. Second-order rings and reflections are also distinguishable. Using the method developed in^8^, a radially integrated normalized intensity profile is displayed in Fig. 2d and summarizes the observed reflections. In the latter, the Cu (111) planes are noticeable at 2.08 Å, which arise from the several nanoparticles seen around the core–shell particle of Fig. 2a (top left and bottom right corners). Second-order reflections of the Gr (002) planes (dotted blue line) are also present (see also Fig. S1b). Note that the Gr (002) planes lead to a large peak with a tail for the greater d_002_ values. This result agrees with HRTEM observations identified for the external shell, which showed a lesser degree of graphitization, i.e., turbostratic (TS) graphite^20^. Finally, the broad peak observed between 1.0 and 1.3 Å is a convolution of the second-order Cu (111) and Gr (101) with third-order Gr (002) bands. Two other examples of similar particles are provided in Fig. S1c,d.

**Figure 3:**
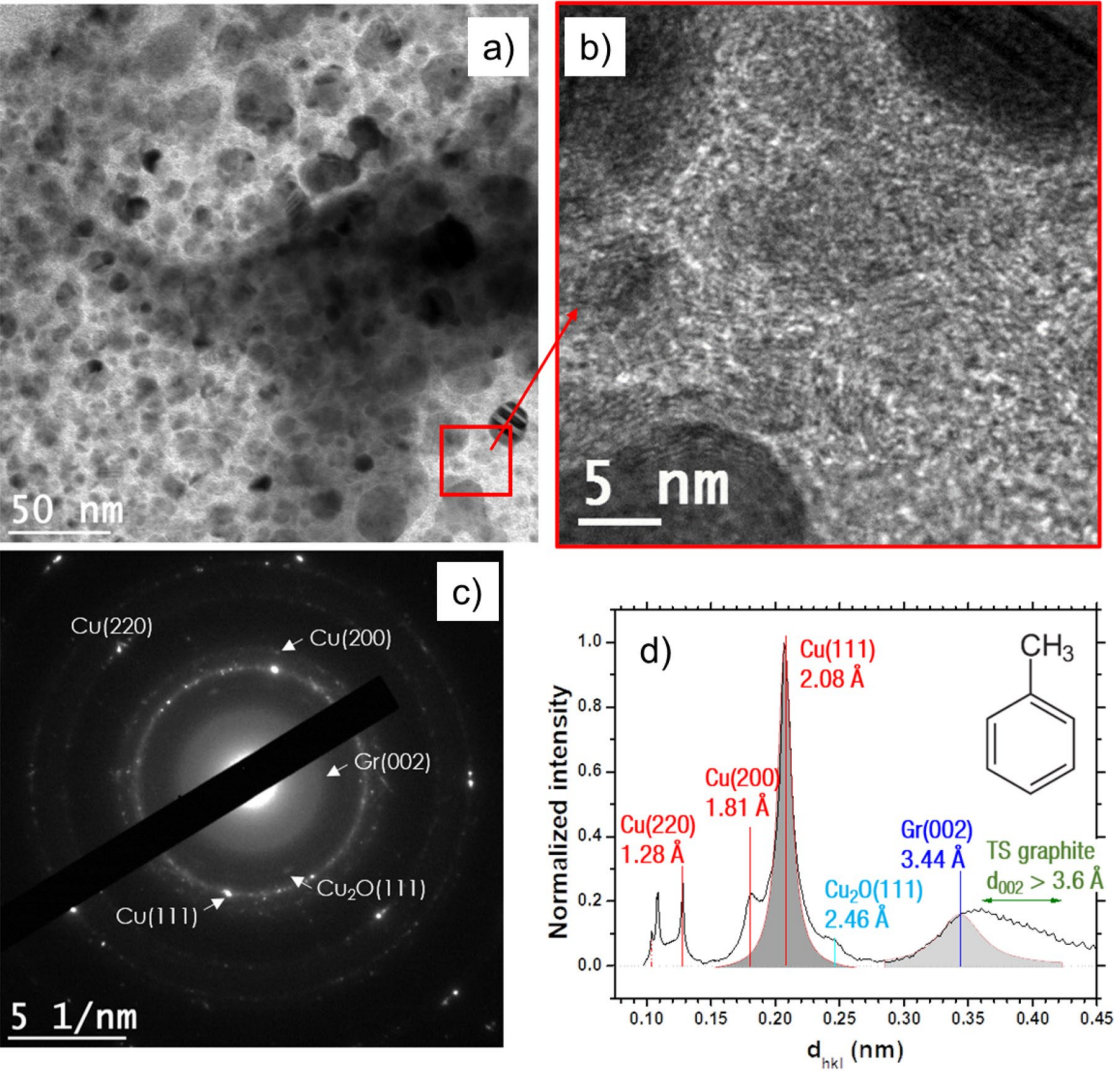
(**a**) High-resolution image of Cu nanoparticles embedded in a carbonaceous matrix synthesized by discharges in toluene. (**b**) HRTEM image of the red inset of (**a**) showing Cu nanoparticles (black) and nanocrystalline carbon. (**c**) SAED pattern from (**a**) radially integrated into (**d**), which mainly indicates pure Cu features with a nanocrystalline graphite signature.

Along with submicrometric particles, clusters of Cu NPs embedded in a carbonaceous matrix are shown in Fig. 3a. Note that the NPs are observed over a hole in the TEM grid carbon film to avoid any contribution from its amorphous phase. Thus, the HRTEM image presented in Fig. 3b shows that the carbon matrix is composed of numerous graphite-like nanocrystals, which is confirmed by the diffraction pattern observed in Fig. 3c where a diffuse Gr (002) ring is observed. The main contribution in the diffraction pattern arises from the Cu (111) and Cu (200) planes from which the many orientations lead to two distinct rings, demonstrating that the vast majority of nanoparticles of Fig. 3a are pure copper. The low oxygen content observed in the EDS spectrum (Figure S1e) confirms this assessment. Other minor reflections are observed in Fig. 3c for cuprite (Cu_2_O (111) at 2.46 Å), but the absence of a diffraction ring reveals the scarcity of the oxide content in the sample. Finally, a few Cu (220) reflections may be observed. The reflections form a ring that is only visible using a contrast-enhancing filter (see Figure S1f. of the supplementary data) because the relative intensity of the Cu (220) reflections is four times lower than the relative intensity of the Cu (111) reflections^21^. The radial integration (Fig. 3d) summarizes and confirms the SAED results. The radial integration features a very large band for disorder/TS graphite and shows the second-order of the copper reflections (~ 1.0–1.1 Å). However, it does not show the reflections from the Gr (002) planes reflecting, once again, the poor degree of graphitization of the carbon phase.

**Figure 4:**
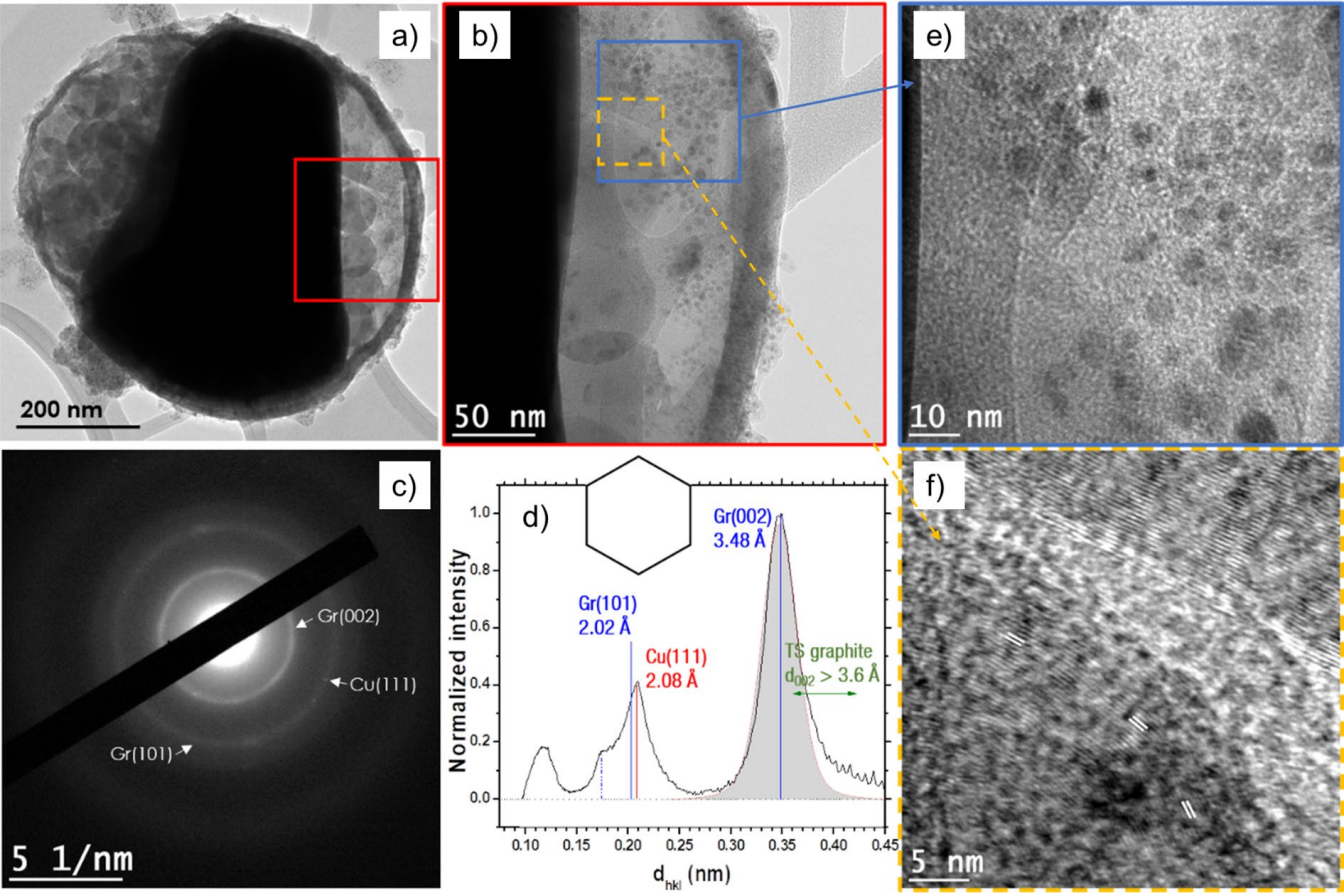
(**a**) High-resolution image of a submicronic particle synthesized by discharges in cyclohexane. (**b**) HRTEM image of the red inset of (**a**), which displays Cu nanoparticles (blue inset, (**e**)) and graphite onions (dashed orange inset, (**f**)). (**c**) SAED pattern from (**a**) exhibiting Cu and Gr rings. (**d**) A radially integrated profile permits to highlight Cu (111) reflections. The dotted blue line corresponds to the second-order graphite (004) reflections.

We have also conducted analogous analysis for particles synthesized by discharges in cyclohexane. Submicronic Cu-Gr particles are observed (Fig. 4a), and, similarly, the core exhibits a non-spherical geometry, while the shell indicates a perfect sphere. A higher magnification presented in Fig. 4b, and its insets highlight the presence of carbon onions^22^ and Cu-bearing NPs. Carbon onions are most likely confined within the shell, whereas the NPs are expected to lie at the surface (as seen in the SEM images in Fig. 1). Moreover, the tweaking of the focus that was needed to obtain a clear image (blue inset) of the nanoparticles also supports this hypothesis. Carbon onions are also observed on the left part of the inner shell, as shown in Fig. 4a. The onion graphite interplanar distance is about 3.85 Å (see white lines, parallel to basal planes, orange inset). Such a distance gives clues regarding the conditions of the synthesis of such structures, excluding the formation of a carbon onion at high pressure^22,23^.

Figure 4c–d depict the SAED pattern of (a). First, the Gr (002) integrated reflections’ center and width are greater than those obtained with toluene, which is explained by the presence of the graphitic shell, the carbon matrix surrounding the Cu NPs, and the carbon onions; these latter exhibit higher interplanar d_002_ spacings. Note that, once again, the mean inner shell d_002_ (~ 3.4 Å) is smaller than that of the outer shell (~ 3.8 Å). Cu (111) and Cu (200) rings stand out, evidencing the presence of multiple Cu nanoparticles. Nonetheless, no cuprite reflections are noticeable, as also confirmed by the absence of oxygen in the EDS spectrum (Figure S2a).

Lastly, the broadband observed around 1.0–1.3 Å is ascribed to second-order Cu (111) and third-order Gr (002) reflections, whereas the bump at 1.7 Å (blue dashed line) is linked with the second-order of the Gr (002) peak. Another example of such core–shell particles may be found in Figure S2c. Additionally, TEM analysis of the NPs synthesized in cyclohexane, similar to that shown in Fig. 3 for toluene, is presented in the supplementary data (Fig. S3).

**Figure 5:**
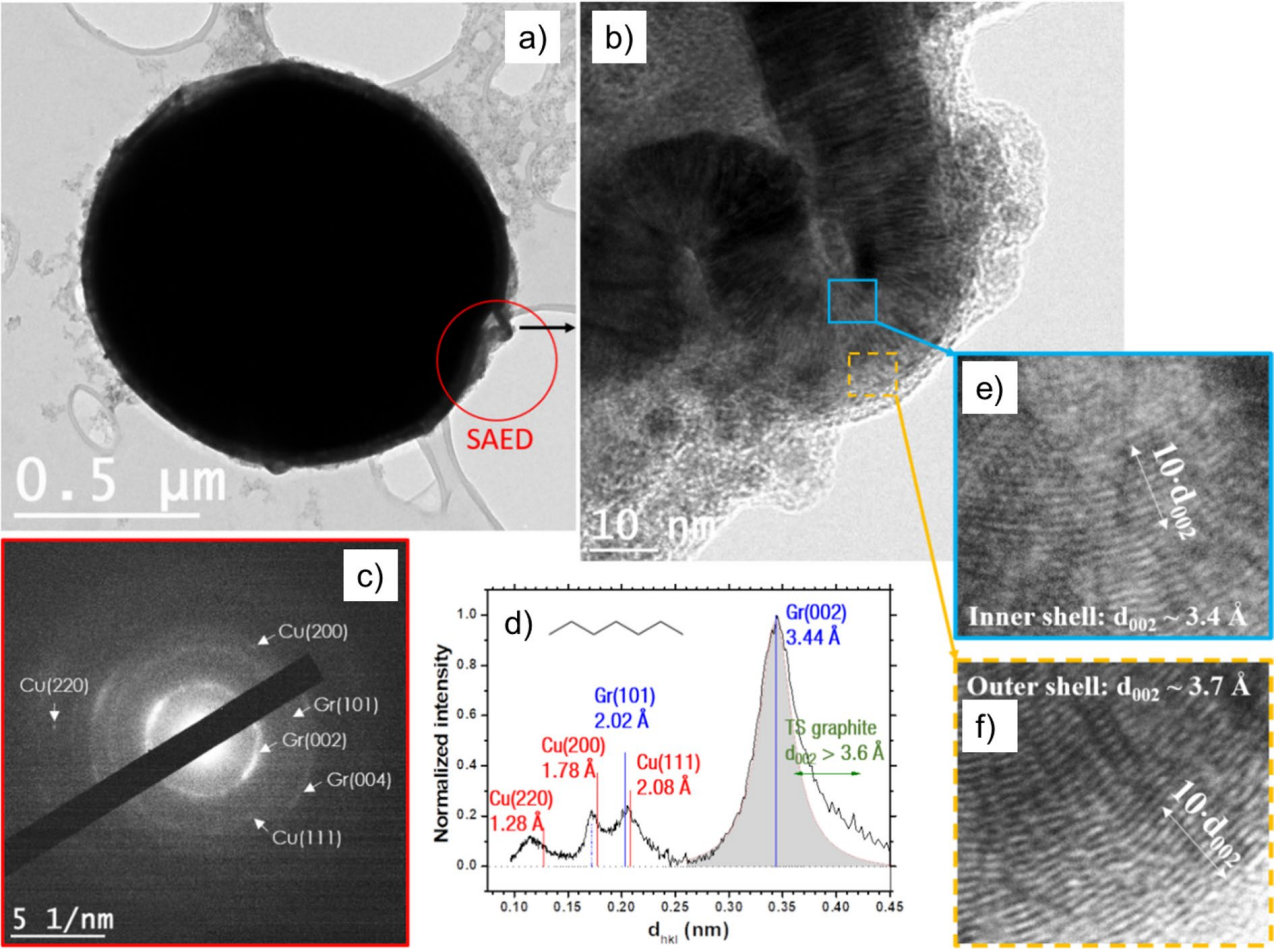
(**a**) High-resolution TEM image of a copper microscopic particle synthesized by discharges in heptane. (**b**) Higher magnification of (**a**) pointed by the black arrow. The insets present the inner (**e**) and outer (**f**) shell d_002_. (**c**) SAED pattern from (**a**) exhibiting Cu (200) reflections and Gr (002) and (100) rings. (**d**) A radially integrated profile permits to highlight Cu (111) reflections. The dotted blue line corresponds to the second-order Gr (002) reflections.

Finally, a similar analysis is conducted for the particles synthesized by spark discharges in heptane. A Cu-Gr microparticle is presented in Fig. 5a. Figure 5b displays a higher magnification, highlighting a fold in the graphitic layers as well as Cu NPs lying on the shell surface. The blue and dashed orange insets expose the discrepancies between the inner and outer layers of the shell. The former has a mean d_002_ of about 3.4 Å (Fig. 5e), while the latter exhibits typical TS graphite features with many dislocations and a d_002_ of around 3.7 Å (Fig. 5f). While the number of observed core–shell particles is low, this shell feature (d_002,inner_ < d_002,outer_) is seemingly observed in every condition; thus, this feature might be associated with the core–shell particle formation mechanism in the spark discharge. Figure 5c presents a SAED pattern of the circled area in (a), with its associated radial integration shown in Fig. 5d. The main features are the Gr (002) and Gr (101) peaks at 3.44 and 2.02 Å, respectively. Note the presence of Cu NPs appearing as single reflections in Fig. 5c, notably Cu (111), Cu (200), and Cu (220) at 2.08, 1.78, and 1.27 Å, respectively. Significant contributions from TS graphite (second- and third-order (002) features) are also observed. To complete this analysis, an EDS spectrum, a contrast-enhanced SAED pattern, and another core–shell particle found within the TEM grid are provided in the supplementary data (Figure S4a–c).

**Figure 6:**
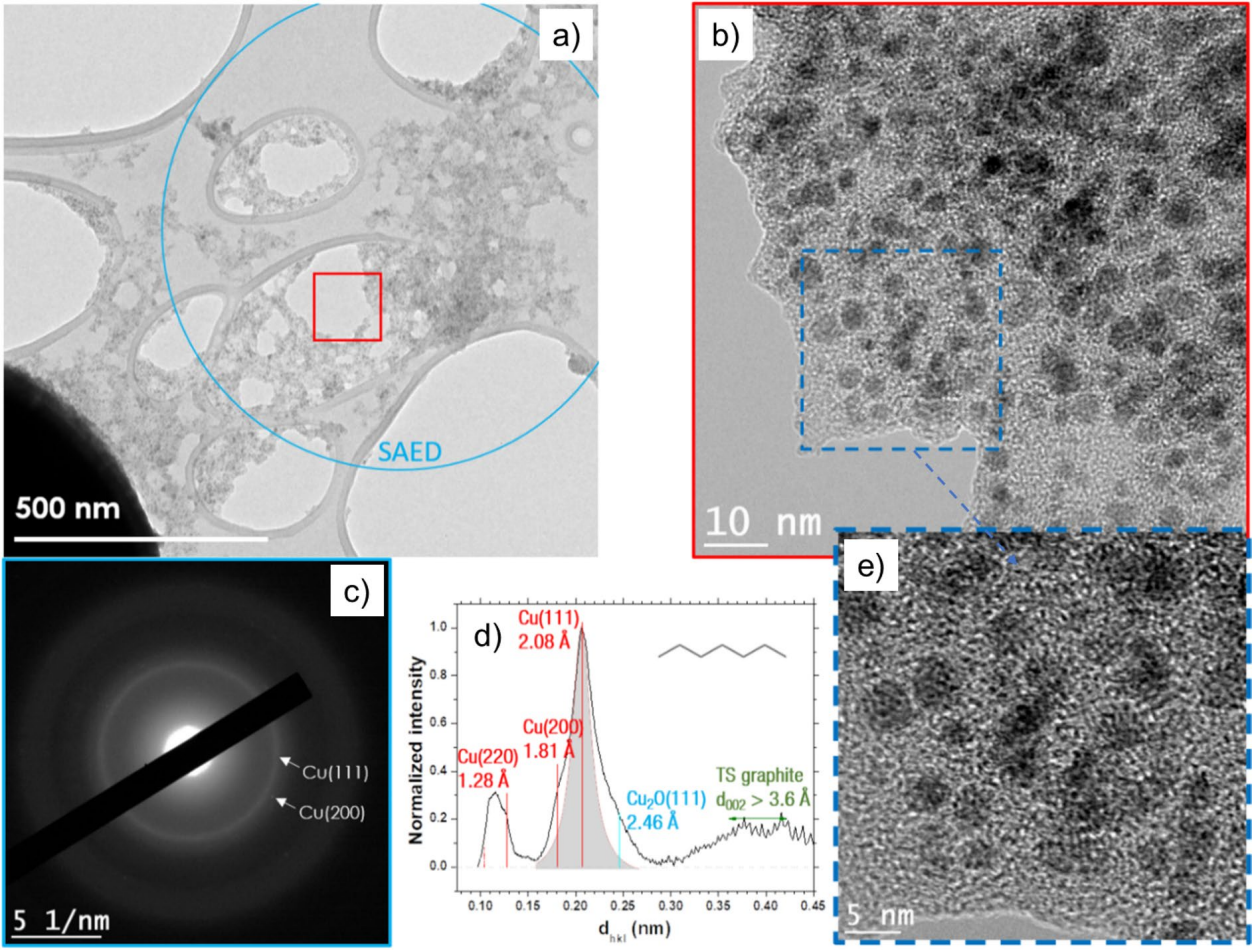
(**a**) High-resolution image of Cu nanoparticles embedded in a carbonaceous matrix synthesized by discharges in heptane. (**b**) HRTEM image of the red inset of (**a**) showing Cu nanoparticles (black) and nanocrystalline carbon (**e**) is a magnification of the dashed blue square in (**b**). (**c**) SAED pattern from (**a**) radially integrated into (**d**). The Cu (111) integrated band is fitted in gray to highlight the presence of a faint cuprite Cu_2_O (111) signature.

We also found numerous patches of Cu NPs embedded in a carbon matrix throughout the grid. The TEM analysis related to one of these patches is presented in Fig. 6. Similarly to Fig. 3, discharges in heptane are characterized by the formation of smaller Cu NPs (Figs. 6b) with a slightly higher cuprite content than those seen in other HC solutions (as presented in Fig. 6c, the EDS shown in Figure S4d, and the contrast-enhanced SAED in Figure S4e) and a much deteriorated degree of graphitization of the carbon matrix—close to an amorphous state—as seen in the dashed inset.

**Figure 7:**
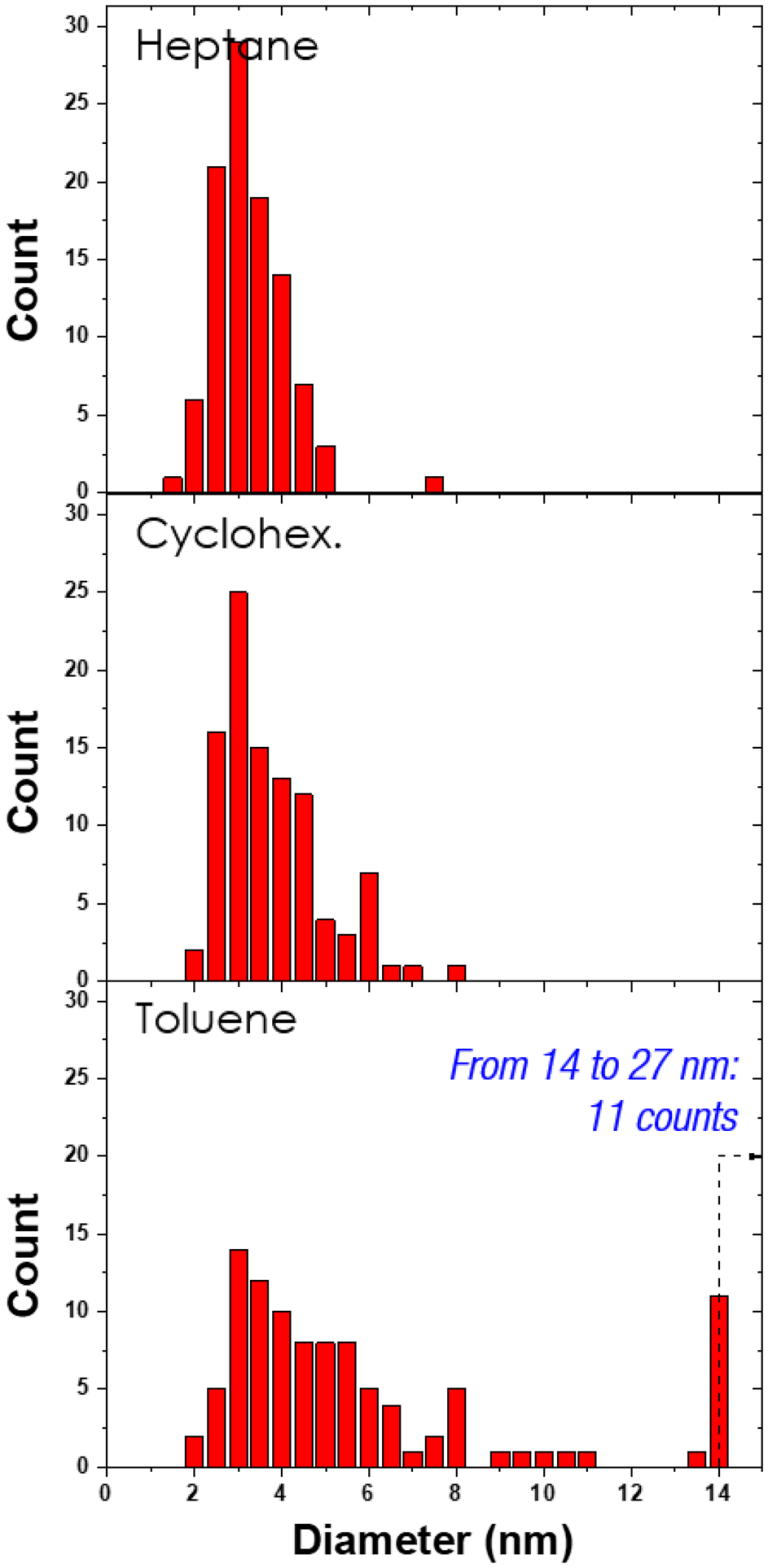
Distribution of the Cu NPs embedded in carbonaceous matrices (n = 100 particles for each condition).

Finally, to determine the size distribution of the NPs synthesized in each condition, we performed a statistical analysis, and the results are shown in Fig. 7. Discharges in heptane produce the sharpest distribution of NPs, in a range of 2–6 nm and a peak at 3 nm, which is even narrow than that reported in the literature^16–18^. NPs synthesized in cyclohexane and toluene have slightly broader range of distributions, from 2 to 8 nm and from 2 to 14 nm, respectively.

## Discussion

### The formation of Cu NPs

It is common to find spherical NPs resulting from discharges in liquids^6,8,24,25^. Due to the heat transferred from the plasma to the electrode, atoms evaporate from the electrode’s surface and form NPs in the plasma zone as a result of species coalescence. In our cases, Cu NPs are observed embedded in a carbon matrix, which is explained by the dissociation of the liquid HCs to release carbonaceous species into the plasma. Under every condition investigated in the present study, we found that Cu NPs are embedded in a carbon matrix with various degree of graphitizations depending on the HC solution used.

On the other hand, the SAED analysis reveals that the degree of graphitization of the carbon matrix is highest with toluene (Fig. 3d) and lowest with heptane (Fig. 6d). The cuprite content is also demonstrated to be higher in heptane than in toluene, which can be related to the exposure of the sample to ambient air and the more porous (amorphous carbon) matrix in heptane. These may expose the Cu particles to the oxidative ambient environment, unlike the denser matrix synthesized in the case of toluene. At this stage, the nature of the liquid HC seems to directly influence the degree of graphitization of the carbon matrix as well as the oxidation of Cu NPs embedded in the matrix. This ‘tune’ of the nanocomposite film characteristics is a finding of interest for specific applications, as the oxidation degree of the NPs (and their properties, *e.g.,* band gap energy) can be finely tuned depending on the utilized liquid.

Considering that the NPs are formed in the plasma phase^8,26^, the gas heat capacity of the various HCs may explain the result. Indeed, the three liquids exhibit different heat capacity values (e.g. at 1500 K, it is 444^27^, 367^28^, and 301 J mol^–1^ K^–1^^29^ for heptane, cyclohexane, and toluene, respectively; although the plasma temperature is sevral thousands of Kelvin, these values at 1500 K are given to compare the heat capacity of the three liquids at high temperature). Thus, for a similar discharge energy, the gas temperature is expected to be the highest for toluene, enabling easier carbonization and an enhanced formation of graphite nanocrystals, as seen in Fig. 3b. Moreover, because the ionization energy (IE) of toluene (IE_toluene_ = 8.82 eV) is the smallest of the three HCs (IE_cyclohexane_ = 9.88 eV and IE_heptane_ = 9.91 eV), the plasma volume is expected to be higher with toluene than those with the other HC solutions, which would lead to an increased residence time of the NPs within the gas phase. This may explain the broader range of the size distribution of the Cu NPs found in toluene, as compared with the other HC solutions. This claim needs further confirmation by measuring, for example, the plasma temperature and volume under these conditions, which will be the topic of another study.

### The formation of Cu-Gr particles and carbon onions

The different core–shell particles presented in this manuscript (and in the supplementary data) share a common peculiarity, i.e., although the graphitic envelope is often spherical, this is not the case for the core Cu particles. More generally, the shell has a perimeter far larger than the perimeter needed to circumvent the core particle. We believe that this discrepancy is the result of two distinct phenomena (detailed below).

At this stage, it is worth remembering that the heavier particles are not synthesized within the plasma phase (like the NPs) but instead are ejected from the electrode’s surface after being affected by the plasma. The discharge has an average temperature of several thousand Kelvin^30^, and, therefore, the surface of the electrode material facing the plasma is expected to be in a superheated liquid phase^6^. Under these conditions and due to the intense pressure gradient arising from the discharge, ejected microparticles are known to have a significant velocity and temperature^6^. Under our conditions, each ejected hot particle surpasses the plasma zone and reaches the liquid phase, where cooling leads to carbonization and graphitization^31^, creating the C-shell. The strong thermal gradient resulting from the discharge may explain both (1) the discrepancies in degree of graphitization previously reported between the inner and outer shell (Figs. 3b, 6b), and (2) the non-spherical shape of the Cu core (see Figs. 3a, 5a, 6a, and supplementary data). Additionally, Cu and Gr exhibit highly different thermal expansion coefficients (α): α _graphite_ ranges from − 1.3 × 10^–6^ K^–1^ at 300 K to 1.1 × 10^–6^ K^–1^ at 1300 K^32^, while α_copper_ monotonously increases from 17 × 10^–6^ K^–1^ at 300 K^33^ to 26 × 10^–6^ K^–1^ at 1300 K^34^. Thus, the Cu microparticle core shrinks more than the C-shell after solidification, which is a feature that might enhance the shape discrepancies observed between core and shell and especially the presence of folds within the shell (Figs. 3c, 6b).

Secondly, while the cooling of hot, fast-traveling microparticles in liquid HC explains the formation of the graphitic shell and the degree of graphitization gradient found within the shell, it does not fully account for the formation of carbon nano-onions. The size of these nanostructures is surprisingly constant, ranging between 80 and 90 nm. This might exclude the formation by the collection of carbon vapor around a C_60_ seed^30,35^, which would lead to a broader size distribution. The formation of nano-onions, as well as a larger-than-the-core separated shell, can be explained by the presence of vortices during flow separation (detachment of the flow from the surface of the object). Such a phenomenon is commonly reported in fluid dynamics within the wake of a flow (Reynolds number, *Re* > 10) passing a spherical object^36–38^. In this *flow past a spheroid* fluid dynamics problem (see Figure S5), the ratio between the shell and the particle sizes (when the thermal shrinkage is neglected) enables the determination of *Re.* This latter is defined as *Re* $= \rho uL/\mu$, where $\rho$ and *μ* are the HC density and dynamic viscosity, respectively, *L* is the particle diameter and *u* the fluid velocity (relative to the particle). Assuming that the HC solution is at ambient temperature and atmospheric pressure, with $\rho$ = 779 kg m^–3^ and $\mu$ = 1 × 10^–3^ Pa s, the particle velocity with respect to a static fluid can be estimated, once *Re* is known. Additionally, considering that vortices and flow separation appear around *Re* = 24^39^, it leads to an estimation of the particle velocity around $u$ = 72 m s^−1^. The real value is obviously not trivial to assess, owing to the non-spherical shape of the particle and the assumptions made. More generally, the determination of a precise value would require a complex model that combines thermodynamics, fluid dynamics, and plasma physics, which is beyond the scope of this study. Nonetheless, the observation of nano-onions strongly implies the existence of vortices at the nanoscale and may improve the understanding of flow separation at the micro/nano-scale. Such phenomena are of particular interest regarding, for example, the study of microscale ocean geology^40^. This is especially true considering that current experimental techniques such as micro-Particle Image Velocimetry^41^ and microscale synthetic schlieren^42^ are limited to particle diameters above 10 to 150 µm, which are far larger than the particles observed in our study.

### Reflection on the graphitic shells

Regarding the C-shell encircling the microparticles, the degree of graphitization of the shell formed in toluene has been observed to be the greatest. We report an integrated (002) peak centered around 0.337 nm (Fig. 2d), while the shells formed in heptane and cyclohexane show values around 0.344 and 0.348 nm for graphite d_002_, respectively. Note that these values are not trivial to analyze since the SAED carried out in Fig. 4 (cyclohexane) also contains numerous onions with a weak degree of graphitization (d_002_ = 0.385 nm), which increases the average measured interplanar spacing. However, we believe that the number of core–shell particles found on the TEM grids is statistically too small to conclude on a link between the observed C-shell properties and the HC solution used. Indeed, degree of graphitization and the number of layers of the shells should not only depend on the features of the liquid (e.g., heat capacity, density) and the plasma (e.g., plasma volume, gas temperature), but also on the initial velocity (amplitude and direction) and temperature of the Cu particle ejected from the electrode. Further understanding of the formation kinetics of the core–shell particles requires additional investigations to report the influence of the liquid composition as well as the size, composition, and velocity/temperature of the particles.

## Conclusion

Sustaining spark discharges in liquid between two copper electrodes immersed in hydrocarbon liquid (toluene, cyclohexane, or heptane) produce two types of Cu-based materials: Cu NPs embedded in a carbon matrix and submicrometric Cu particles encapsulated in C-shell. The shape and size distribution of the latter particles were found to be independent of the hydrocarbon liquid. However, the Cu NPs and carbonaceous matrices in which they are embedded depend on the nature of the utilized liquid. Indeed, the nanoparticles synthesized in toluene are embedded in a highly graphitized carbon matrix, whereas the matricies of the NPs produced in heptane and cyclohexane are characterized by lower degrees of graphitization. The difference is ascribed to variations in the heat capacities of hydrocarbon gaseous phases. The size distribution of the Cu NPs is also dependent on the nature of the liquid. The smallest particles are produced in heptane. On the other hand, some of the Cu microparticles with C-shell have carbon nanoonions trapped between the core and the shell. The presence of these particles suggests that micrometric vortices may present during the ejection of hot Cu particle in liquid. To further understand the formation of these peculiar shells, complex multidisciplinary models that take particle shape, particle size, and number of graphitic layers into account must be developed.

## Materials and methods

### Experimental setup and protocol

**Figure 8:**
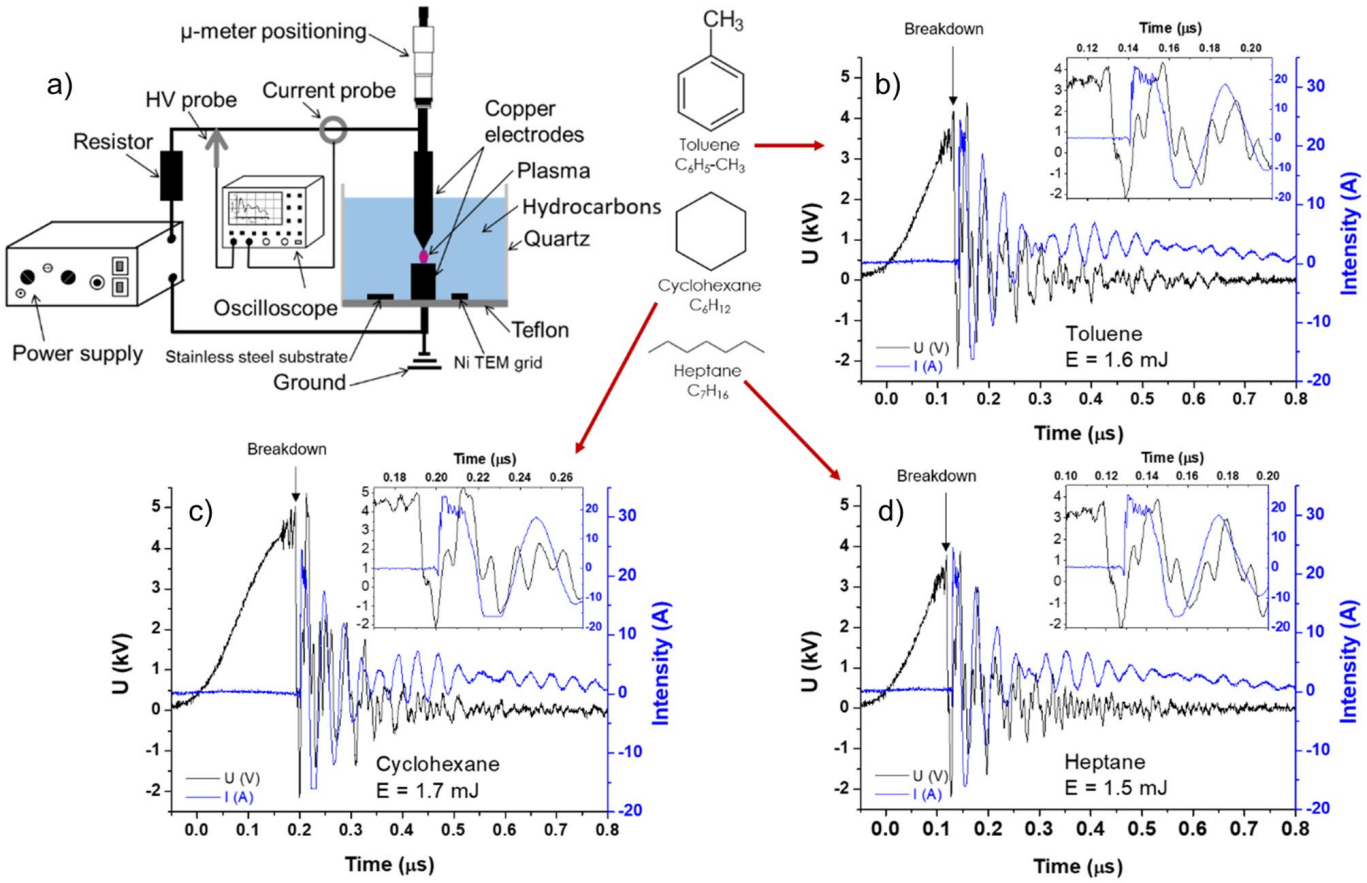
(**a**) Schematic of the experimental setup used to produce spark discharges between two copper electrodes immersed in various liquid HCs: toluene, cyclohexane, and heptane. (**b**–**d**) The I–V characteristics of a typical discharge in each condition are also displayed. The insets highlight the evolutions of the I–V curves after the breakdown.

The experimental setup (Fig. 8) used here is similar to the one extensively described in a previous work^8^. Briefly, the reactor walls are made of a quartz cylinder (inner diameter of 5 cm) and a Teflon base. Spark discharges are initiated between two copper rods (purity of 99.95% and diameters of 2 mm). The top electrode (anode) is mechanically polished to have a needle-like tip (apex angle ~ 35°) mounted on a translational stage allowing fine horizontal and vertical positioning, while the cathode has a flat top (i.e., pin-to-plate configuration). The sparks are generated using a high-voltage power supply (Cober Electronics Inc., model 606) at an amplitude of ~ 4 kV and a pulse-width of ~ 2 µs, at a repetition rate of 70 Hz. The voltage (V) and current (I) are measured using a high-voltage probe (Tektronix, model P6015a) and a wideband terminated current transformer (Lilo, LTD, model 13w5000), respectively. I–V waveforms are visualized and recorded throughout the experiment using an oscilloscope (Tektronix, DPO 5204B). The discharges are consistent in terms of the voltage and current shown in Fig. 8, with an average energy of about 1.5 mJ per pulse. Since the discharges induce electrode erosion, the anode position is continuously adjusted to keep a similar inter-electrode gap distance.

As a preparation step for each experimental case, the apparatus is thoroughly cleaned using acetone, then methanol, and finally, the relevant HC solution. Three liquid HCs were used: toluene (C_6_H_5_–CH_3_), cyclohexane (C_6_H_12_), and n-heptane (C_7_H_16_). These liquids were chosen to investigate the effect of different molecular structures (aromatic, cyclic or linear, shown in Fig. 8) and H/C ratios.

Figures 8b–d present typical I–V characteristics of the discharge in the different HC solutions. Changing the HC does not strongly affect either the behavior of the I–V curves or the discharge energy. The breakdown voltage oscillates between 3.5 and 4.5 kV, demonstrating no distinctive difference among tested HCs. The inset of each graph, magnifying the moment of the breakdown, clearly supports the insignificant dependence of I–V on the properties of the chosen liquids. The mean spark energy for all conditions is about 1.54 mJ (toluene averages 1.51 mJ, cyclohexane 1.54 mJ, and heptane 1.57 mJ).

### Ex-situ diagnostics

Each spark induces electrode erosion, and the eroded species are injected both in the plasma and the liquid. Sample particles generated by the discharges are collected on a stainless steel (SS) substrate and a Ni C-lacey grid located at the bottom base (Fig. 8). The conductive SS substrate is used for scanning electron microscopy (SEM, JEOL JSM6700F at 3 kV), while the Ni grid allows for transmission electron microscopy (TEM, JEOL JEM-2100F at 200 kV). Under a 200-kV beam, electrons transfer up to 43 eV to the graphite lattice^19^, which is greater than its threshold displacement energy^43,44^. Incident electrons thus lead to the formation of numerous Frenkel pairs. Therefore, to limit the damage generated by the electron beam, fine-tuning of the image is performed at a location away from the target location, and final snapshots are quickly taken at the desired location. The beam current is also decreased when possible. Selected area electron diffraction (SAED) is always carried out before high-resolution (HRTEM) imaging to assess the crystalline order of the particles to avoid any beam-induced damage. The SAED patterns are analyzed using a radial integration technique based on the use of an ImageJ plugin to generate the *Radial Profile Plot*^45^. Briefly, the plugin integrates the pixel intensities of the diffraction pattern over concentric circles of different radii, defined from the center of the diffraction pattern, and the integrated value is divided by the number of pixels forming each respective circle. Following the work of Schamp and Jesser^46^, the error in the SAED measurement has been estimated at 2%. Also, we note that the use of space-averaged characterization techniques, such as Raman spectroscopy or X-ray diffraction, are not helpful because of the non-homogeneity of the particles.

## Supplementary Information

Supplementary Information

## Data Availability

The datasets generated during and/or analyzed during the current study are available from the corresponding author on reasonable request.
