# Supplementary material for: Synthesis of core–shell copper–graphite submicronic particles and carbon nano-onions by spark discharges in liquid hydrocarbons
Source: Sci Rep. 2021 Apr 6;11:7516. doi: 10.1038/s41598-021-87222-x (PMC8024313; doi:10.1038/s41598-021-87222-x)
Supplement: Supplementary file 1 — Supplementary Information [file 41598_2021_87222_MOESM1_ESM.docx]

**SUPPLEMENTARY INFORMATION**

**Synthesis of Core-Shell Copper-Graphite Submicronic Particles and Carbon Nano-Onions by Spark Discharges in Liquid Hydrocarbons**

X. Glad^1^, J. Gorry^1^, M. S. Cha^2^ and A. Hamdan^1,*^

^1^Groupe de physique des plasmas, Département de Physique, Université de Montréal,

1375 Avenue Thérèse-Lavoie-Roux, Montréal, H2V 0B3, Québec, Canada.

^2^Physical Science and Engineering Division (PSE), Clean Combustion Research Center (CCRC), King Abdullah University of Science and Technology (KAUST), Thuwal 23955, Saudi Arabia

*corresponding author. Email: [ahmad.hamdan@umontreal.ca](mailto:ahmad.hamdan@umontreal.ca)

***Figure S1.***


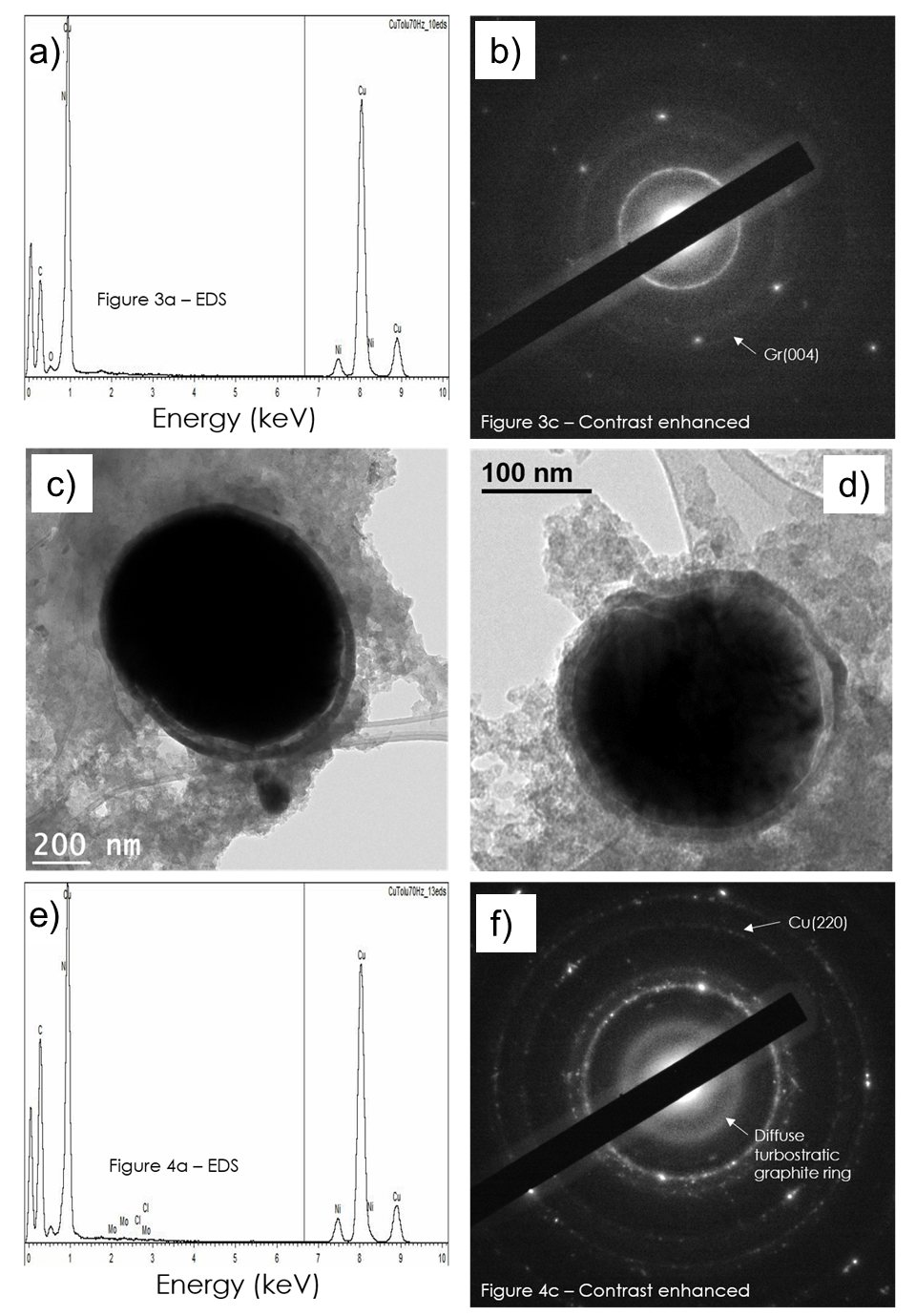


***Figure S1.* Toluene**: (a) EDS spectrum and (b) contrast-enhanced SAED patterns (using ImageJ’s *Enhanced Local Contrast* filter) of **Figure 2** of the manuscript. (c,d) display two other examples of core-shell Cu-Gr particles found within the TEM grid. (e) EDS spectrum and (f) contrast-enhanced SAED pattern associated with **Figure 3** of the manuscript. The contrast-enhanced SAED patterns reveal SAED contributions that were too dim to be observed by the naked eye. Of particular interest, one can now discern the second order of the graphite (002) planes (subfigure b) as well as the Cu(220) ring and the boundaries of the diffuse turbostratic graphite ring (subfigure f). Regarding the EDS spectra, Ni peaks are present because the TEM grid is made out of nickel.

***Figure S2.***


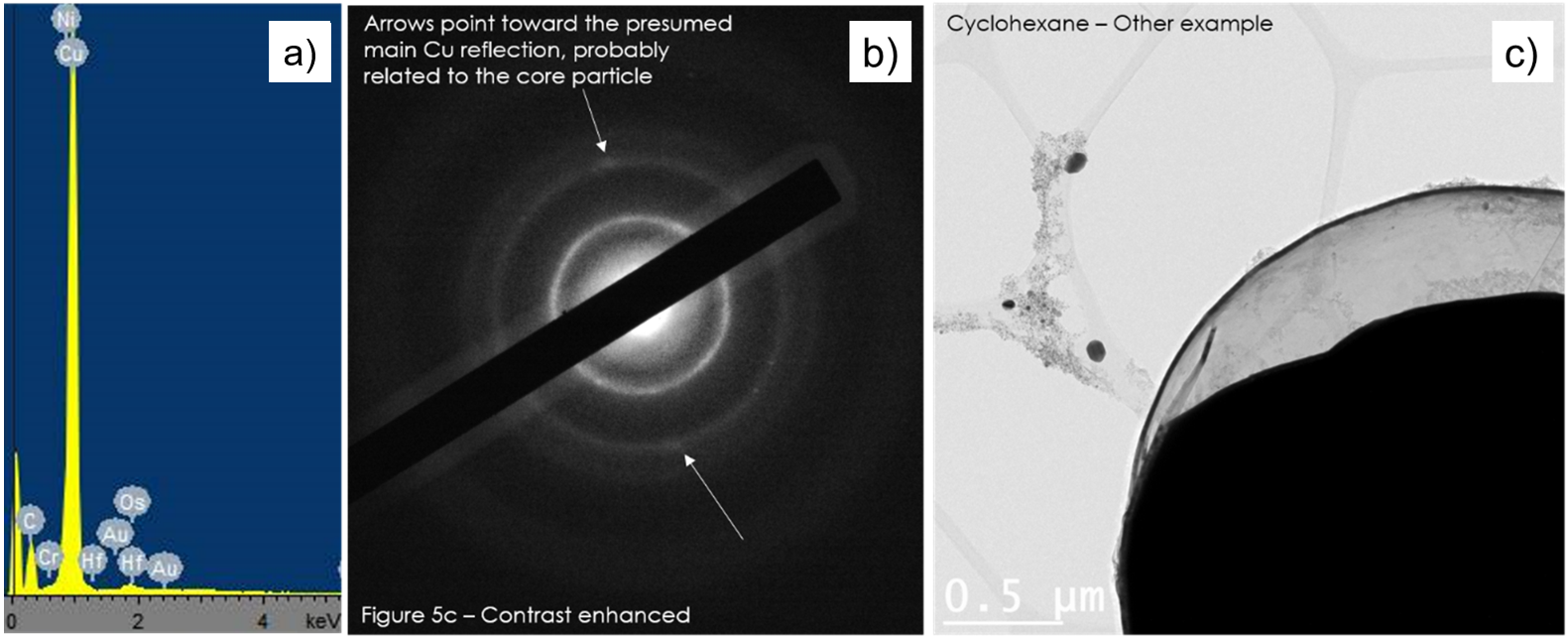


***Figure S2.* Cyclohexane:** (a) EDS and (b) contrast-enhanced SAED pattern of **Figure 4**. (c) shows another core-shell Cu-Gr particle.

***Figure S3.***


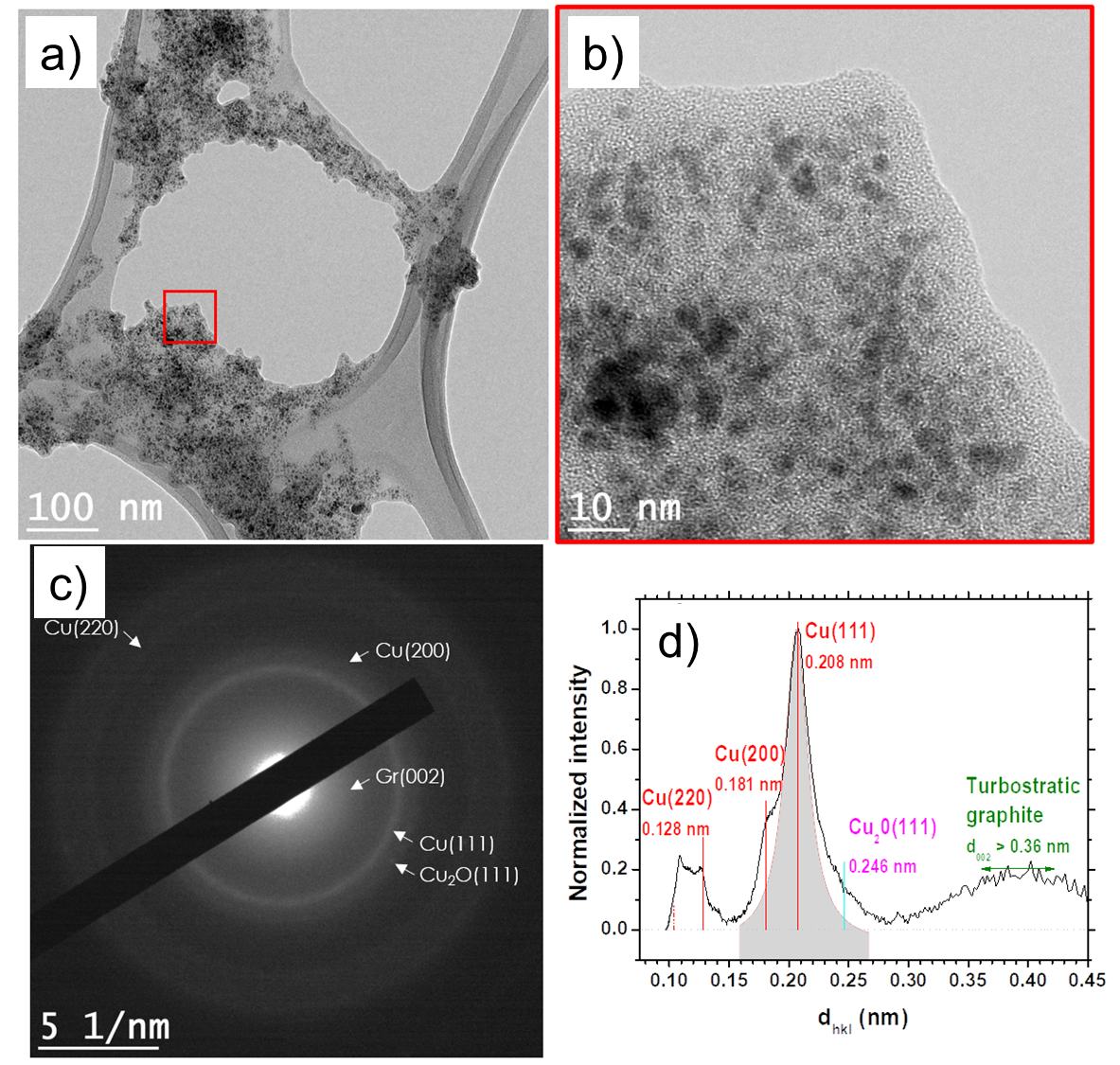


***Figure S3.* Cyclohexane:** (a) High-resolution image of Cu nanoparticles embedded in a carbonaceous matrix synthesized by spark discharges in cyclohexane. (b) HRTEM image of the red inset of (a) showing Cu nanoparticles (black) and nanocrystalline carbon. (c) SAED pattern from (a) radially integrated into (d). The Cu(111) integrated band is fitted in gray to highlight the faint presence of a cuprite Cu2O(111) signature.

***Figure S4.***


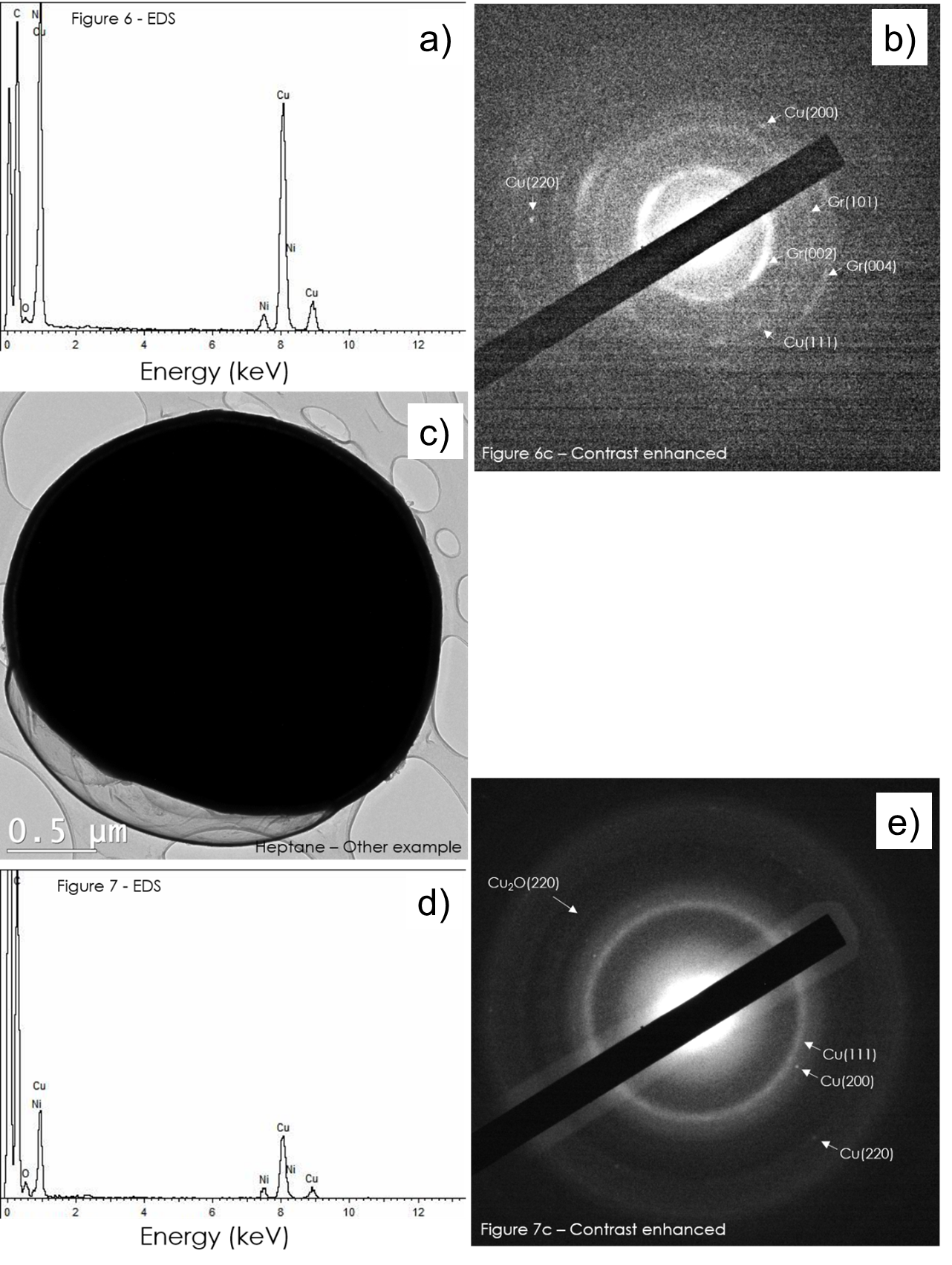


***Figure S4.* Heptane:** (a) EDS spectrum and (b) contrast-enhanced SAED patterns of **Figure 5** of the manuscript. (c) displays another example of a core-shell Cu-Gr particle found within the TEM grid. (d) EDS spectrum and (e) contrast-enhanced SAED pattern associated with **Figure 6** of the manuscript. A faint cuprite (220) ring (~ 0.15 nm) may be observed in the latter.

***Figure S5.***


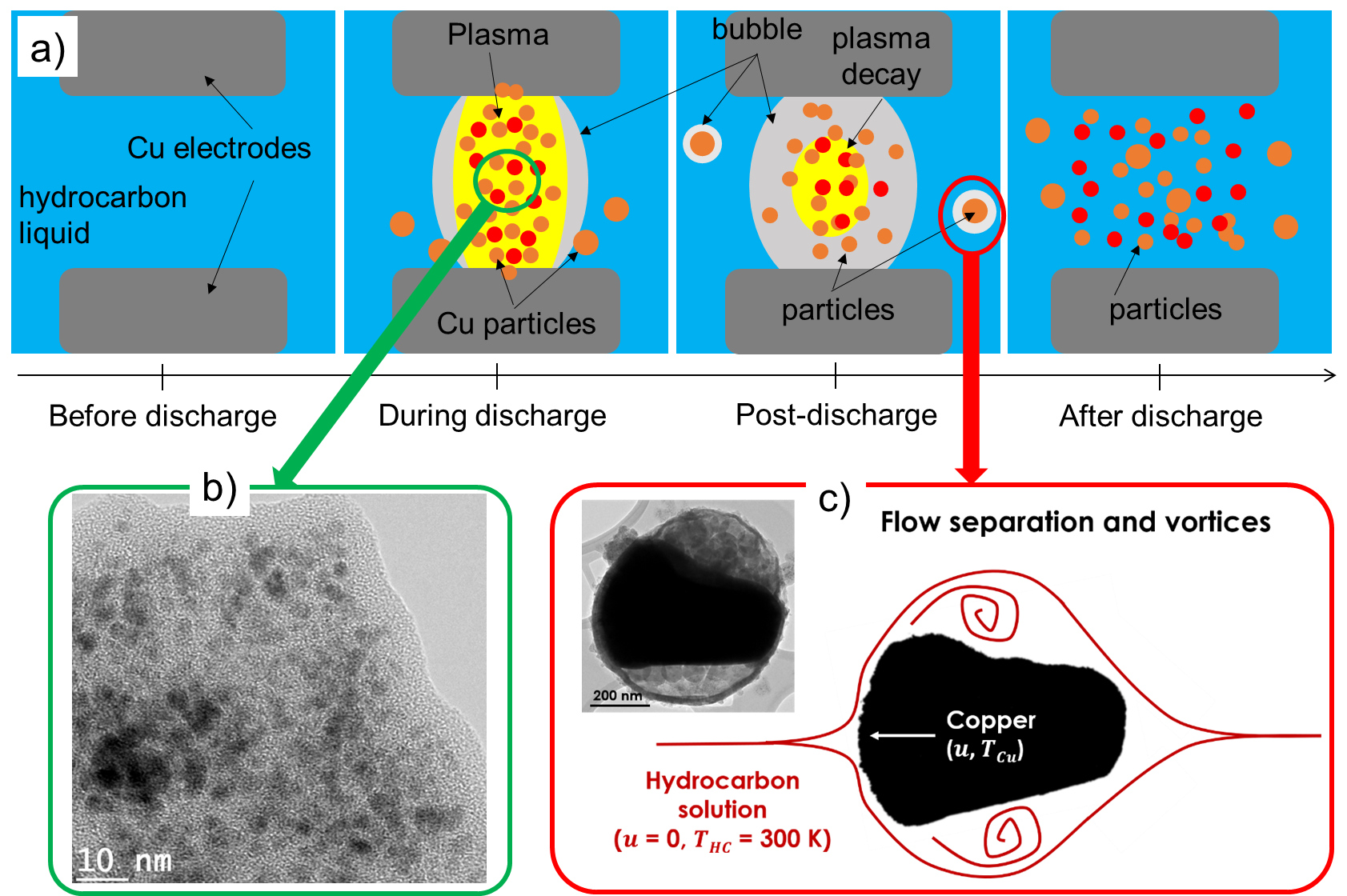


***Figure S5.*** (a) Simplified sketch illustrating the mechanisms during discharge that lead to the formation of nanocomposite material (b) in the plasma core and core-shell particle (c) of copper due to ejection of hot particles in liquid. The formation of carbon onions within the carbon shell can be explained by flow separation and vortices. The carbon onions form at the vortices location. Note that this sketch is a simplified representation only; no simulation has been made.
